# The early onset of peripheral neuropathy might be a robust predictor for time to treatment failure in patients with metastatic breast cancer receiving chemotherapy containing paclitaxel

**DOI:** 10.1371/journal.pone.0184322

**Published:** 2017-09-12

**Authors:** Ippei Fukada, Yoshinori Ito, Kokoro Kobayashi, Tomoko Shibayama, Shunji Takahashi, Rie Horii, Futoshi Akiyama, Takuji Iwase, Shinji Ohno

**Affiliations:** 1 Department of Breast Medical Oncology, Breast Oncology Center, the Cancer Institute Hospital of the Japanese Foundation for Cancer Research, Tokyo, Japan; 2 Department of Medical Oncology, the Cancer Institute Hospital of the Japanese Foundation for Cancer Research, Tokyo, Japan; 3 Department of Pathology, the Cancer Institute Hospital of the Japanese Foundation for Cancer Research, Tokyo, Japan; 4 Department of Pathology, the Cancer Institute of the Japanese Foundation for Cancer Research, Tokyo, Japan; 5 Department of Breast Surgical Oncology, Breast Oncology Center, the Cancer Institute Hospital of the Japanese Foundation for Cancer Research, Tokyo, Japan; 6 Breast Oncology Center, the Cancer Institute Hospital of the Japanese Foundation for Cancer Research, Tokyo, Japan; University of North Carolina at Chapel Hill School of Medicine, UNITED STATES

## Abstract

**Background:**

Paclitaxel plays a central role in chemotherapy for breast cancer. Peripheral neuropathy, a well-known toxicity with paclitaxel, may be of interest in predicting the efficacy of paclitaxel therapy for patients with metastatic breast cancer. We performed a retrospective analysis assessing whether the early occurrence of peripheral neuropathy (EPN) was a predictive marker for better efficacy in patients with metastatic breast cancer receiving chemotherapy containing paclitaxel.

**Patients and methods:**

Between January 2000 and August 2008, we examined the records of 168 patients with metastatic breast cancer treated with paclitaxel in our hospital. EPN was defined as a symptom of Grade 2 or more during first three months of treatment. The overall response rate (ORR) and time to treatment failure (TTF) in each group were analyzed retrospectively.

**Results:**

Of 168 patients with metastatic breast cancer who were treated with paclitaxel, EPN was documented in 101 patients (60.1%). The clinical benefit rate (CR, PR, and SD ≥ 6 months) was 72.3% in the EPN group and 49.3% in the non-EPN group (p = 0.002). The TTF of the EPN group (median 11.2 months, 95% CI: 9.5–12.9) was significantly longer than that of the non-EPN group (5.7 months, 95% CI: 4.6–6.8) (p<0.001). Multivariate analysis demonstrated that EPN (p<0.001), dose intensity of less than 70% (p<0.001), and the history of microtubule agents (p = 0.001) were the significant favorable prognostic factors for TTF.

**Conclusion:**

The early onset of peripheral neuropathy might be a robust predictor for TTF in patients with metastatic breast cancer treated with paclitaxel.

## Introduction

Taxane drugs binds to β-tubulin, promotes microtubule polymerization, and prevents depolymerization, thereby arresting cell division and inducing apoptosis. In Japan, paclitaxel was approved for breast cancer in 1999. Currently, paclitaxel plays a central role in chemotherapy for breast cancer. Although with this treatment, neuropathy is a common toxicity, there have been many reports of the therapeutic effects of paclitaxel for metastatic breast cancer. For instance, a randomized phase III trial compared the effects of docetaxel and paclitaxel on metastatic breast cancer, reporting that the overall response rate (ORR) and median time to progression (TTP) for paclitaxel were 25.0% and 3.6 months, respectively [1]. The incidence of all grade and grade 3/4 neurosensory toxicities were 59.0% and 4.1% in the paclitaxel groups. Furthermore, the incidence of all grade and grade 3/4 neuromotor toxicities were 12.6% and 2.3% in the paclitaxel groups. It was also reported that the RR and median TTP for paclitaxel 175 mg/m2 intravenously as the first-line therapy were 19% and 16.9 weeks, respectively [2]. Treatment-related grade 3 sensory neuropathy occurred in 2% of the paclitaxel arm. Seidman et al. reported the final results of randomized phase III trial of weekly compared with every-3-weeks paclitaxel for metastatic breast cancer, with trastuzumab for all HER-2 overexpressors and random assignment to trastuzumab or not in HER-2 nonoverexpressors (Cancer and Leukemia Group B protocol 9840). In their study, the incidence of grade 2 and grade 3 neurosensory toxicity were 33% in patients who received constant dosing of paclitaxel at 175 mg/m2 q3w, 45% in 80 mg/m2 weekly arm, and 51% in 100 mg/m2 weekly arm, respectively [3]. Commonly, peripheral neuropathy is managed by dose reduction and treatment delays. There have been no reports as to whether peripheral neuropathy may be predictive of the efficacy of paclitaxel therapy for patients with metastatic breast cancer.

We performed a retrospective analysis assessing whether the early occurrence of peripheral neuropathy constituted a predictive marker for improved efficacy in patients with metastatic breast cancer who had received chemotherapy containing paclitaxel.

## Material and methods

### Patients and methods

Of 527 patients with metastatic breast cancer treated in our hospital between January 2000 and August 2008, 168 who were treated with paclitaxel were included. Comprehensive consent for the use of specimen materials was obtained by written informed consent from all patients participating as subjects in this study. The retrospective study was approved by the Institutional Review Board of the Cancer Institute Hospital of the Japanese Foundation for Cancer Research (2013–1136), and data were collected in compliance with the ethical requirements of our institution.

Immunohistochemical subtypes were determined by biopsy of the primary lesion at stage IV or surgical materials of primary tumor in patients with recurrent breast cancer. They were stained with hematoxylin-eosin (HE) and were immunohistochemically examined for ER, PgR, and HER2. Immunohistochemical assessment of ER and PgR expression was performed using antibodies for ER, clone 1D5 (Dako Japan Inc., Tokyo, Japan) and for PgR, clone PgR636 (Dako Japan Inc.). Positive reactions for ER and PgR were defined as nuclear staining in 10% or more of cancer cells, and negative reactions were defined as staining in less than 10%. Hormone receptor positivity was defined as showing positivity in ER and/or PgR.

Immunohistochemical detection of HER2 protein was performed using the Hercep Test (Dako Japan Inc.). Expression of HER2 protein was classified into four groups: 0, 1+, 2+, and 3+. In those cases that were 2+, HER2 genetic testing by FISH was performed using a PathVysion HER2-DNA Probe Kit (Abbott Molecular Inc., Des Plaines, IL). Both protein and genetic status were estimated based on the guidelines for HER2 testing in breast cancer, as edited by the American Society of Clinical Oncology/College of American Pathologists [4]. HER2 positivity was defined as HER2 protein 3+ or HER2 gene amplification.

After combining ER, PgR, and HER2, patients were classified into four subtypes, defined as follows: luminal subtype, ER+ and/or PgR+, HER2-; luminal HER2 subtype, ER+ and/or PgR+, HER2+; HER2 subtype, ER-, PgR-, HER2+; and triple negative subtype, ER-, PgR-, HER2-.

### Treatment

The chemotherapy regimens given to patients in this study included a weekly paclitaxel (wPAC) regimen at a dose of 80 mg/m^2^. Concurrent trastuzumab was used for all patients of the luminal-HER2 type and HER2 type (loading dose of 4 mg of intravenous trastuzumab per kilogram of body weight, followed by 2 mg per kilogram weekly).

### Dose intensity

The intensity of the paclitaxel dose was analyzed at the standard regimen of paclitaxel, 80 mg/m^2^ once a week. Relative dose intensity (RDI) was the ratio of actual total dose intensity (ATDI) and planned total dose intensity (PTDI), expressed as a percentage and calculated as follows:

RDI(%)=ATDI/PTDI×100

PTDI was the dose intensity planned for the entire treatment duration, averaged across the chemotherapy agents used. ATDI was defined as the actual average dose intensity over real treatment duration. Cumulative dose was the same as the actual dose.

### Toxicity and definition of early onset of peripheral neuropathy

Patients who received a paclitaxel regimen at a dose of 80 mg/m^2^ visited our hospital every week, and physicians recorded their clinical symptoms in detail. The neuropathy information have been retrospectively collected by first author based on clinic notes, and toxicity was assessed according to the National Cancer Institute Common Terminology Criteria for Adverse Events version 4.0 (NCI-CTC-AE v4). Based on this criteria, we carefully checked clinic notes retrospectively. The moderate symptoms; limiting instrumental ADL, severe symptoms; limiting self care ADL, and symptoms which needed dose reduction and administration of neuroprotective agents were defined as the symptoms of Grade 2 or more. Finally, the early onset of peripheral neuropathy (EPN) was defined as symptoms of Grade 2 or more during the first three months of treatment.

### Response evaluation and statistical analysis

Radiological tumor assessments were performed by computed tomography every 3–4 months during treatment. Local lesion (breast, chest wall, and skin) and regional lymph node metastases were measured by echogram. The response to chemotherapy was assessed according to the Response Evaluation Criteria in Solid Tumors (RECIST) version 1.1. The overall response rate (ORR) was defined as the proportion of patients achieving complete response (CR) and partial response (PR). The clinical benefit rate (CBR) was defined as the proportion of patients with CR, PR, and stable disease (SD) lasting >6 months. Time to treatment failure (TTF) was used as the surrogate marker for the efficacy of paclitaxel. TTF was defined as time interval from the start of paclitaxel treatment to disease progression or cessation of treatment because of adverse events. SPSS ver. 17.0 was used for statistical analysis in this study. ORR and CBR were analyzed using the χ^2^ test. TTF was measured by the log-rank test using the Kaplan-Meier method. For multivariate analysis, Cox regression analysis was used. A p-value of less than 0.05 was considered statistically significant.

## Results

### Patient characteristics

Characteristics of all 168 patients are listed in Table 1. All evaluated cases had invasive breast cancer. The median age was 56 years (29–84). Patient numbers according to age group age were as follows: <39 years old, 20 patients (11.9%); >40 years old, 148 patients (88.1%). The most common sites of distant metastasis were the lymph nodes, found in 102 patients (60.7%). The mean number of metastases was three. The number of patients receiving one or more prior treatment was 132 (78.6%); 72 patients (42.9%) had a history of receiving anthracyclines.

**Table 1:** Patient characteristics and EPN distribution.

	non-EPN		EPN		p value
	n = 67	%	n = 101	%	
Age					
median	53.0		57.0		
range	29–76		29–84		
Age distribution					
≧50	41	24.4	78	46.4	0.037
≦49	26	15.5	23	13.7	
Status					
Stage IV	25	14.9	24	14.3	0.058
Postoperative recurrence	42	25.0	77	45.8	
DFI (days)	1444.5		1293.0		
Number of metastatic sites					
median	3		3		
range	1–6		1–7		
Site of metastasis					
Lymph node	40	23.8	62	36.9	0.827
Liver	35	20.8	43	25.6	0.219
Lung	32	19.0	53	31.5	0.550
Bone	46	27.4	59	35.1	0.179
Brain	19	11.3	20	11.9	0.198
Local	15	8.9	21	12.5	0.805
(breast, chest wall, skin, regional LN)					
Breast	13	7.7	12	7.1	0.180
Number of prior chemotherapies					
None	14	8.3	22	13.1	0.961
1	23	13.7	37	22.0	
2	14	8.3	22	13.1	
3	10	6.0	11	6.5	
4≦	6	3.6	9	5.4	
History of anthraxycline					
For metastasis	28	16.7	44	26.2	0.820
Neoadjuvant or Adjuvant	18	10.7	31	18.5	0.593
History of microtubule agents	12	7.1	11	6.5	0.252
Subtype					
Luminal	31	18.5	51	30.4	0.483
Luminal-HER2	9	5.4	11	6.5	
HER2	14	8.3	27	16.1	
Triple negative	13	7.7	12	7.1	

EPN, early onset of peripheral neuropathy; DFI, disease free interval; LN, lymph node; NAC, neoadjuvant chemotherapy; HER2, human epidermal growth factor receptor type2; IHC, immunohistochemistry; FISH, fluorescence *in situ* hybridization; RDI, relative dose intensity

### Early onset of peripheral neuropathy and clinicopathological features of each group

In our study, EPN appeared in 101 patients (60.1%). Associations between EPN and the distribution of clinicopathological features are shown in Table 1. Although there was significant difference between age among two groups, there was no important differences between subtype distribution, portions of metastasis, number of prior chemotherapy treatments, or history of anthracycline and microtubule agents.

### Cumulative dose

Cumulative dose of all 168 patients are shown in Table 2. Median cumulative dose was 1720.0 mg/m^2^ in all patients. Median cumulative dose was 1120.0 mg/m^2^ in the non-EPN group and 1920.0 mg/m^2^ in the EPN group (p <0.001).

**Table 2:** Cumulative dose, dose intensity and response rate.

	Non-EPN n = 101	EPN n = 67	p-value
Cumulative dose			
Median (mg/m^2^)	1120.0	1920.0	<0.001
Dose intensity			
Median (mg/m^2^)	60.1	49.2	
Range (mg/m^2^)	24.3–80.0	24.2–80.0	
Median RDI (%)	76.0	61.5	<0.001
Response rate			
CR	0 (0%)	3 (1.8%)	
PR	24 (14.3%)	38 (22.6%)	
SD	18 (10.7%)	51 (30.4%)	
Long SD	9 (5.4%)	32 (19.0%)	
PD	25 (14.9%)	9 (5.4%)	
ORR (%)	35.8 (%)	40.6 (%)	0.488
CBR (%)	49.3 (%)	72.3 (%)	0.002
CBR according to subtypes			
Luminal	35.5%	62.7%	
Luminal-HER2	77.8%	77.8%	
HER2	38.5%	58.3%	
Triple negatibe	71.4%	85.2%	

RDI, relative dose intensity; CR, complete response; PR, partial response; SD, stable disease; Long SD, SD lasting >6 months; PD, progressive disease; ORR, overall response rate; CBR, clinical benefit rate.

### Dose intensity and relative dose intensity

In all patients, dose intensity was 52.3 mg/m^2^ (range 24.2–80.0 mg/m^2^) and RDI was 66.4% (30.2–100%). The dose intensity and RDI were 60.1 mg/m^2^ (range 24.3–80 mg/m^2^), 76.0% (30.4–100%) in the non-EPN group and 49.2 mg/m^2^ (range 24.2–80 mg/m^2^), 61.5% (30.2–100%) in the EPN group (p <0.001) (Table 2).

### Response rate

ORR and CBR for each group are shown in Table 2. The ORR was 40.6% in the EPN group and 35.8% in the non-EPN group. CBR was 72.3% in the EPN group and 49.3% in the non-EPN group. While there was no important difference in ORR within each group, there was a significant difference in CBR (p = 0.002). For each immunohistochemical subtype, CBR in the EPN and in the non-EPN groups were 62.7% and 35.5% in the luminal type, 100% and 77.8% in the luminal-HER2 type, 85.2% and 71.4% in the HER2 type, and 58.3% and 38.5% in the triple negative type, respectively.

### Time to treatment failure according to subtypes

**Fig 1:**
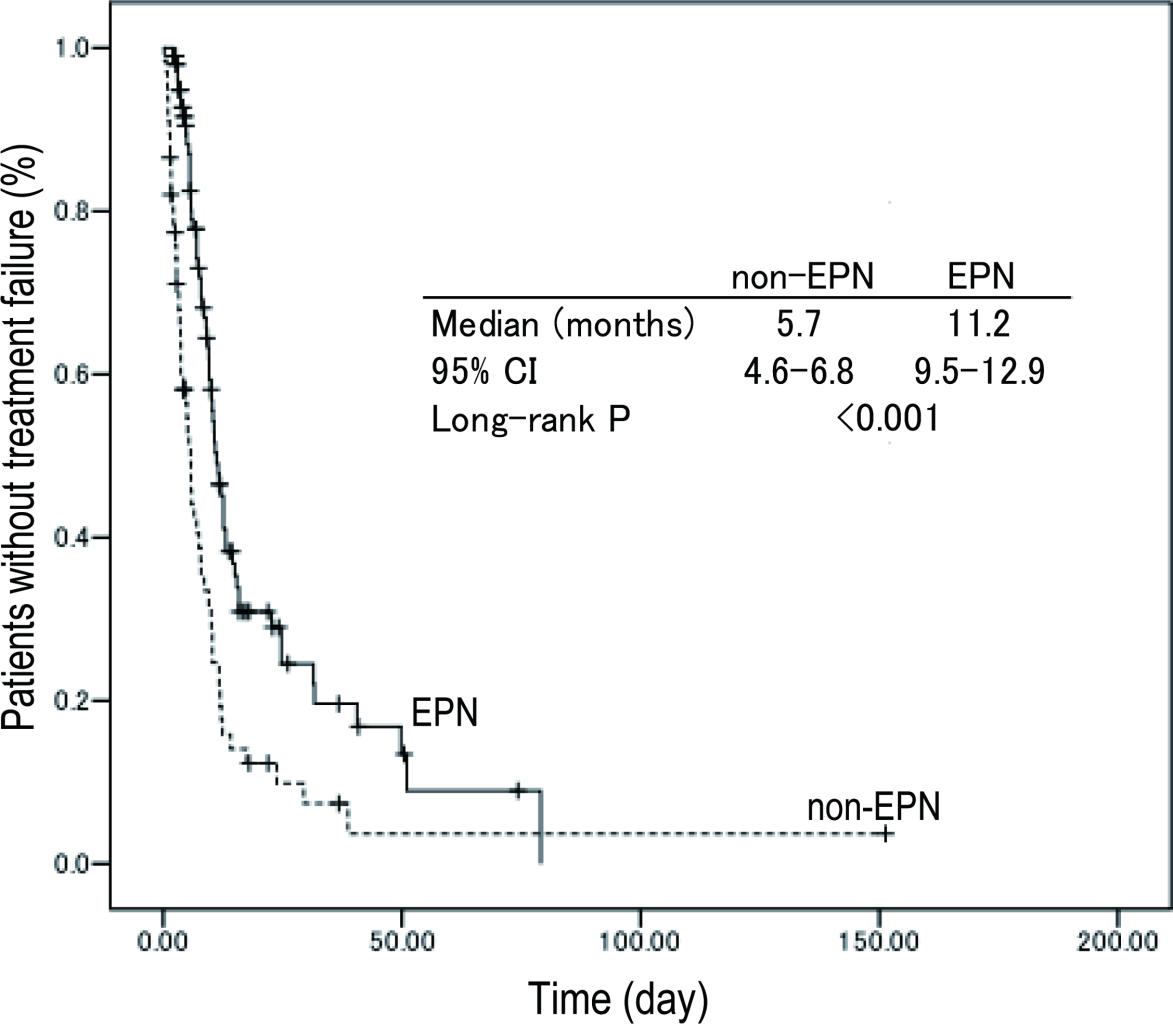
Kaplan-Meier plots for TTF. The TTF of the EPN group (median 11.2 months, 95% CI; 9.15–12.9) was significantly longer than that of the non-EPN group (5.7 months, 95% CI; 4.6–6.8) in all patients.

**Fig 2:**
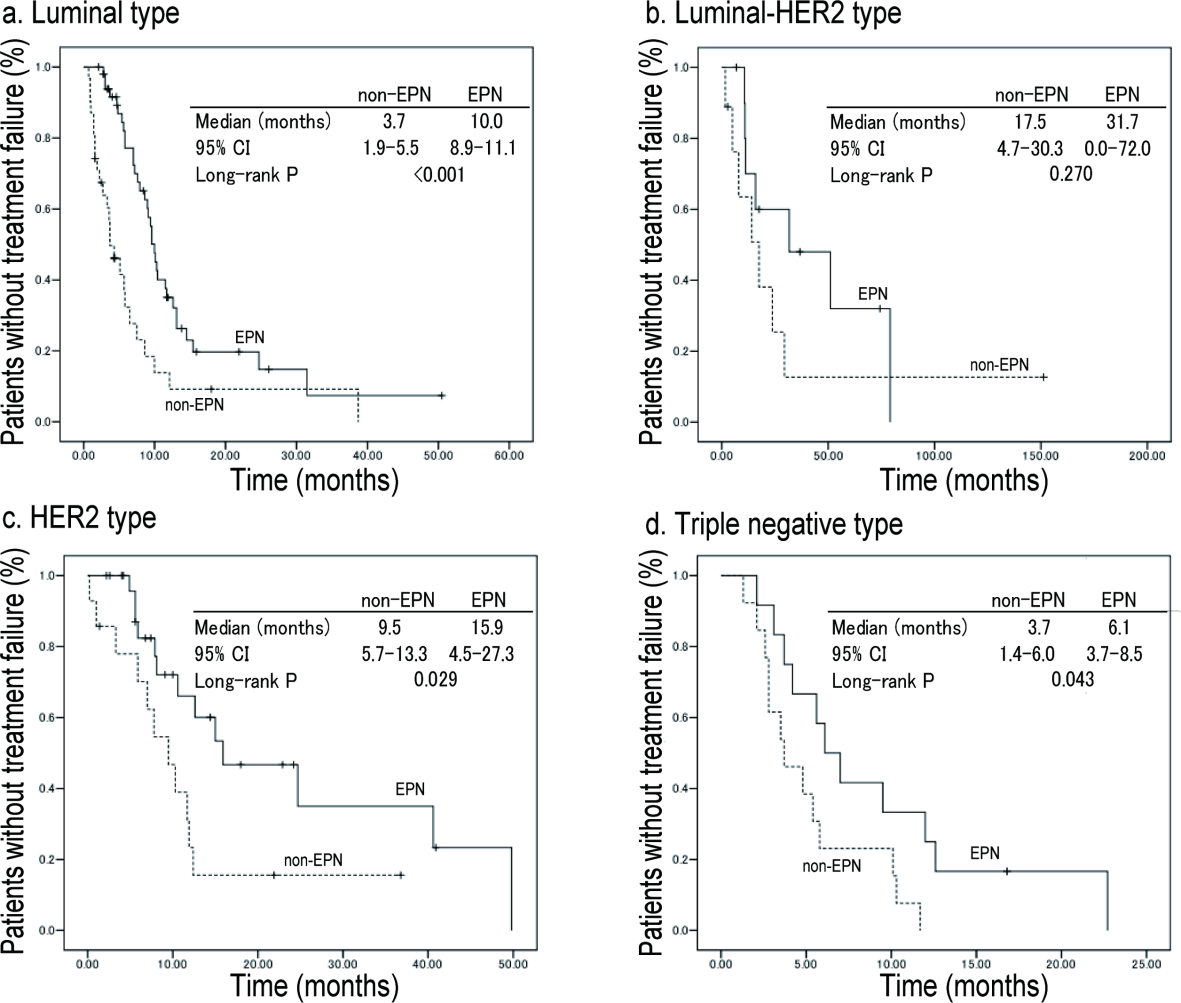
Kaplan-Meier plots for TTF according to subtypes. Among the four subtypes, TTF was 10.0 months in the EPN group and 3.7 months in the non-EPN group in the luminal type (p = <0.001); 31.7 months in the EPN group and 17.5 months in the non-EPN group in the luminal-HER2 type (p = 0.270); 15.9 months in the EPN group and 9.5 months in the non-EPN group in the HER2 type (p = 0.029); and 6.1 months in the EPN group and 3.7 months in the non-EPN group of the triple negative type (p = 0.043).

Average follow-up time was 11.8 months. TTF for the EPN group (median 11.2 months, 95% CI; 9.5–12.9) was significantly better than that of the non-EPN group (5.7 months, 95% CI; 4.6–6.8) in all patients shown in Fig 1. TTF according to subtype was shown in Fig 2.

### Univariate and multivariate analyses of prognostic factors

Univariate and multivariate analyses of prognostic factors are shown in Table 3. In univariate analysis, the significant prognostic factors for TTF were EPN (relative risk [RR], 0.462; 95% CI, 0.322 to 0.662; p<0.001); dose intensity more than 70% (RR, 2.225; 95% CI, 1.532 to 3.231; p<0.001), subtype (RR, 1.008; 95% CI, 0.848 to 1.197; p<0.001), and history of microtubule agents (RR, 2.337; 95% CI, 1.415 to 3.858; p = 0.001). Multivariate analysis demonstrated that EPN (p<0.001), dose intensity less than 70% (p<0.001), and the history of microtubule agents (p = 0.001) were the significant favorable prognostic factors for TTF.

**Table 3:** Univariate and multivariate analysis of prognostic factors.

Factors	Univariate		Multivariate	
	Relative Risk 95% CI	p-value	Relative Risk	P-value
EPN	0.462			
yes vs no	0.322–0.662	<0.001	0.505	<0.001
Age	0.776			
≦49 vs 50≦	0.524–1.148	0.199	1.057	0.789
Dose intensity	2.225			
>70% vs ≦70%	1.532–3.231	<0.001	1.998	0.001
Subtype	1.008			
Luminal vs Luminal-HER2 vs HER2 vs TN	0.848–1.197	<0.001	0.920	0.356
Site of metastases	1.148			
Visceral vs non-visceral	0.727–1.813	0.550		
No of prior therapy	1.328			
>1vs ≦1	0.926–1.905	0.120		
History of microtubule agents	2.337			
yes vs no	1.415–3.858	0.001	2.340	0.001

## Discussion

We previously reported on our retrospective analysis of the therapeutic effect of taxanes on metastatic breast cancer in various immunohistochemical subtypes [5]. We found that the immunohistochemical subtypes were associated with the therapeutic effect of taxanes for metastatic breast cancer (clinical benefit rate and median TTP, 51.6% and 8.3 months in luminal; 78.6% and 14.1 months in luminal-HER2; 71.9% and 10.6 months in HER2; and 40.8% and 4.2 months in triple negative, p<0.001). However, the TTP in each subtype was distributed over a wide range. Further investigation was required to identify the predictive markers associated with taxane therapy for patients with metastatic breast cancer, according to subtype.

In paclitaxel therapy, the main adverse events that disturb treatment include paclitaxel-associated peripheral neuropathy. The mechanism of taxane-induced peripheral neuropathy is related to the presence of disrupted microtubules of the mitotic spindle. This disruption interferes with axonal transport and macrophage activation in both the dorsal root ganglia and peripheral nerve, as well as with microglial activation within the spinal cord [6, 7, 8]. Moreover, taxane induced a dying-back process starting from distal nerve endings followed by effects on Schwann cells and other neuronal cells, which is an essential microtubule-based process that moves cellular components over long distances between neuronal cell bodies and nerve terminals [7].

Peripheral neuropathy may be of interest in predicting the efficacy of paclitaxel therapy for breast cancer patients. In the ECOG 1199 study, the development of neuropathy was found not to be predictive of survival outcome for patients with early operable breast cancer who had received adjuvant taxane therapy [9]. There has been no previous report showing the development of neuropathy to be predictive of survival outcomes in metastatic breast cancer. However, we revealed in this study that EPN occurred in 101 patients (60.1%) and that the early development of peripheral neuropathy strongly associated with prolongation of TTF in patients with metastatic breast cancer. The overall incidence of peripheral neuropathy is similar to that previously reported in phase III studies in patients with MBC and to other published retrospective studies, which ranges from 18–83% [10].

Possible causes of the occurrence of peripheral neuropathy of paclitaxel associated with strong predictor for prolongation of TTF in patients with metastatic breast cancer include the following factors. Peripheral neuropathy, a dose-dependent adverse event, is a major toxicity associated with paclitaxel. The predominant risk factor for peripheral neuropathy is the cumulative dose over time [11]. Moreover, Mielke S et al reported that there is an obvious mechanism for this association. It is well established that higher drug concentrations, a longer duration of systemic concentration above a threshold of 0.05 uM, were associated with greater neuropathy [12]. They also revealed that the effects of time above paclitaxel concentrations of 0.05 micromol/l was associated with response to treatment, and this emphasizes the value of threshold models for the investigation of paclitaxel pharmacodynamics [13]. Our results strongly suggested that patients with higher drug concentrations have greater neuropathy and greater efficacy. The longer the period of treatment, the more the total dose of paclitaxel increases, resulting in more instances of severe peripheral neuropathy in patients receiving paclitaxel-containing chemotherapy. It is crucial to identify prognostic factors other than the total dose. Therefore, in this study, we defined EPN at Grade 2 or more during first three months of treatment as the key symptom. Appropriately reducing the dose of paclitaxel when peripheral neuropathy worsens could make it possible to extend treatment and to gain the maximum therapeutic effect for these patients, and at the same time prolonging TTF.

In our study, age was not a significant prognostic factor for TTF. Older age has been definitively the predictors associated with an increased risk of developing taxane-induced neuropathy. Hershman et al. reported age was independent predictors of the development of chemotherapy-induced peripheral neuropathy; for each increase in age of one year, the odds of neuropathy increased 4% (p = .006) [14]. The results of the Eastern Cooperative Oncology Group (ECOG) 5103 study showed a significant association between neuropathy and age (12.9% increase with each 10 years; p = 0 .004) [15]. In these studies of women with early-stage breast cancer treated with weekly paclitaxel in adjuvant therapy, a genome-wide association study showed that SNPs in two genes, RWDD3 and TECTA, were significantly associated with time to onset of neuropathy. However, age was not associated with an increased risk of neuropathy in the ECOG 1199 study [16]. In our study, there was no significant association between the occurrence of neuropathy and age.

Regarding the pharmacokinetics of paclitaxel, Paclitaxel is metabolized to inactive compounds by CYP2C8 and CYP3A4 in the liver [17, 18, 19]. Hertz et la. Showed that there is a low-activity variant of the main paclitaxel metabolizing enzyme (CYP2C8*3) that has been shown to be associated with increased paclitaxel effectiveness [20] and neuropathy [21]. Although further investigation is required to identify biomarkers, including the pharmacokinetic mechanism that we have uncovered via clinical samples, for the early onset of peripheral neuropathy in metastatic breast cancer treated with paclitaxel, our data shows the important result that the early onset of peripheral neuropathy might help to motivate patients to continue treatment with paclitaxel in clinical practice.

In conclusion, the early onset of peripheral neuropathy of paclitaxel might be a robust predictor for prolonged TTF in patients with metastatic breast cancer.

## Supporting information

S1 Table Clinical information for neoadjuvant chemotherapy dataset. (XLSX)

## References

[1] Jones SE, Erban J, Overmoyer B, Budd GT, Hutchins L, Lower E, et al Randomized phase III study of docetaxel compared with paclitaxel in metastatic breast cancer. J Clin Oncol 2005; 23: 5542–51. doi: 10.1200/JCO.2005.02.027 16110015 10.1200/JCO.2005.02.027

[2] Gradishar WJ, Tjulandin S, Davidson N, Shaw H, Desai N, Bhar P, et al Phase III trial of nanoparticle albumin-bound paclitaxel compared with polyethylated castor oil-based paclitaxel in women with breast cancer. J Clin Oncol 2005; 23:7794–803. doi: 10.1200/JCO.2005.04.937 16172456 10.1200/JCO.2005.04.937

[3] Seidman AD, Berry D, Cirrincione C, Harris L, Muss H, Marcom PK, et al Randomized phase III trial of weekly compared with every-3-weeks paclitaxel for metastatic breast cancer, with trastuzumab for all HER-2 overexpressors and random assignment to trastuzumab or not in HER-2 nonoverexpressors: final results of Cancer and Leukemia Group B protocol 9840. J Clin Oncol 2008; 26: 1642–9. doi: 10.1200/JCO.2007.11.6699 18375893 10.1200/JCO.2007.11.6699

[4] Wolff AC, Hammond ME, Hicks DG, Dowsett M, McShane LM, Allison KH, et al Recommendations for human epidermal growth factor receptor 2 testing in breast cancer. American Society of Clinical Oncology/College of American Pathologists clinical practice guideline update. Arch Pathol Lab Med 2014; 138: 241–56. doi: 10.5858/arpa.2013-0953-SA 24099077 10.5858/arpa.2013-0953-SA PMC4086638

[5] Fukada I, Araki K, Kobayashi K, Kobayashi T, Horii R, Akiyama F, et al Therapeutic effect of taxanes on metastatic breast cancer of various immunohistochemical subtypes. Oncol letters 2016; 12(1): 663–9. 10.3892/ol.2016.4627 PMC4907289 27347197

[6] Argyriou AA, Koltzenburg M, Polychronopoulos P, Papapetropoulos S, Kalofonos HP. Peripheral nerve damage associated with administration of taxanes in patients with cancer. Crit Rev Oncol Hematol 2008; 66(3):218–28. doi: 10.1016/j.critrevonc.2008.01.008 18329278 10.1016/j.critrevonc.2008.01.008

[7] LaPointe NE, Morfini G, Brady ST, Feinstein SC, Wilson L, Jordan MA. Effects of eribulin, vincristine, paclitaxel and ixabepilone on fast axonal transport and kinesin-1 driven microtubule gliding: implications for chemotherapy-induced peripheral neuropathy. Neurotoxicology 2013; 37:231–9. doi: 10.1016/j.neuro.2013.05.008 23711742 10.1016/j.neuro.2013.05.008 PMC4169189

[8] Peters CM, Jimenez-Andrade JM, Jonas BM, Sevcik MA, Koewler NJ, Ghilardi JR, et al Intravenous paclitaxel administration in the rat induces a peripheral sensory neuropathy characterized by macrophage infiltration and injury to sensory neurons and their supporting cells. Exp Neurol 2007; 203(1):42–54. doi: 10.1016/j.expneurol.2006.07.022 17005179 10.1016/j.expneurol.2006.07.022

[9] Schneider BP, Zhao F, Wang M, Stearns V, Martino S, Jones V, et al Neuropathy is not associated with clinical outcomes in patients receiving adjuvant taxanecontaining therapy for operable breast cancer. J Clin Oncol 2012; 30:3051–7. doi: 10.1200/JCO.2011.39.8446 22851566 10.1200/JCO.2011.39.8446 PMC3732004

[10] Rivera E, Cianfrocca M. Overview of neuropathy associated with taxanes for the treatment of metastatic breast cancer. Cancer Chemother Pharmacol 2015; 75(4):659–70. doi: 10.1007/s00280-014-2607-5 25596818 10.1007/s00280-014-2607-5 PMC4365177

[11] Grisold W, Cavaletti G, Windebank AJ. Peripheral neuropathies from chemotherapeutics and targeted agents: diagnosis, treatment, and prevention. Neuro Oncol 2012; 14 Suppl 4:iv45–54. 23095830 10.1093/neuonc/nos203 PMC3480245

[12] Mielke S, Sparreboom A, Steinberg SM, Gelderblom H, Unger C, Behringer D, et al Association of Paclitaxel pharmacokinetics with the development of peripheral neuropathy in patients with advanced cancer. Clin Cancer Res. 2005; 1;11(13):4843–50. doi: 10.1158/1078-0432.CCR-05-0298 16000582 10.1158/1078-0432.CCR-05-0298

[13] Mielke S, Sparreboom A, Behringer D, Mross K. Paclitaxel pharmacokinetics and response to chemotherapy in patients with advanced cancer treated with a weekly regimen. Anticancer Res. 2005; 25: 4423–7. 16334120

[14] Hershman DL, Till C, Wright JD, Awad D, Ramsey SD, Barlow WE et al Comorbidities and Risk of Chemotherapy-Induced Peripheral Neuropathy Among Participants 65 Years or Older in Southwest Oncology Group Clinical Trials. J Clin Oncol 2016; 34: 3014–22. doi: 10.1200/JCO.2015.66.2346 27325863 10.1200/JCO.2015.66.2346 PMC5012713

[15] Schneider BP, Li L, Radovich M, Shen F, Miller KD, Flockhart DA, et al Genome-Wide Association Studies for Taxane-Induced Peripheral Neuropathy in ECOG-5103 and ECOG-1199. Clin Cancer Res. 2015; 21(22):5082–91. doi: 10.1158/1078-0432.CCR-15-0586 26138065 10.1158/1078-0432.CCR-15-0586 PMC4717479

[16] Schneider BP, Zhao F, Wang M, Stearns V, Martino S, Jones V, et al Neuropathy is not associated with clinical outcomes in patients receiving adjuvant taxane-containing therapy for operable breast cancer. J Clin Oncol 2012; 30(25):3051–7. doi: 10.1200/JCO.2011.39.8446 22851566 10.1200/JCO.2011.39.8446 PMC3732004

[17] Harris JW, Rahman A, Kim BR, Guengerich FP, Collins JM. Metabolism of taxol by human hepatic microsomes and liver slices: participation of cytochrome P450 3A4 and an unknown P450 enzyme. 1994; Cancer Res 54: 4026–35. 7913410

[18] Rahman A, Korzekwa KR, Grogan J, Gonzalez FJ, Harris JW. Selective biotransformation of taxol to 6 alphahydroxytaxol by human cytochrome P450 2C8. 1994; Cancer Res 54 (21):5543–46. 7923194

[19] Kumar G, Ray S, Walle T, Huang Y, Willingham M, Self S, et al Comparative in vitro cytotoxic effects of taxol and its major human metabolite 6 alpha-hydroxytaxol. Cancer Chemother Pharmacol. 1995; 36 (2): 129–35. 7767949 10.1007/BF00689197

[20] Hertz DL, Motsinger-Reif AA, Drobish A, Winham SJ, McLeod HL, Carey LA, et. al CYP2C8*3 predicts benefit/risk profile in breast cancer patients receiving neoadjuvant paclitaxel. Breast Cancer Res Treat 2012; 134 (1):401–10. doi: 10.1007/s10549-012-2054-0 22527101 10.1007/s10549-012-2054-0 PMC3727245

[21] Hertz DL, Roy S, Motsinger-Reif AA, Drobish A, Clark LS, McLeod HL, et al CYP2C8*3 increases risk of neuropathy in breast cancer patients treated with paclitaxel. Ann Oncol 2013; 24 (6):1472–8. doi: 10.1093/annonc/mdt018 23413280 10.1093/annonc/mdt018 PMC3660078

